# COVID-19, Vaccination, and Female Fertility in the Czech Republic

**DOI:** 10.3390/ijms231810909

**Published:** 2022-09-18

**Authors:** Lucie Kolatorova, Karolina Adamcova, Jana Vitku, Lenka Horackova, Marketa Simkova, Marketa Hornova, Michala Vosatkova, Veronika Vaisova, Antonin Parizek, Michaela Duskova

**Affiliations:** 1 Department of Steroids and Proteofactors, Institute of Endocrinology, Narodni 8, 116 94 Prague, Czech Republic; 2 Department of Obstetrics and Gynecology, General University Hospital, 1st Faculty of Medicine, Charles University in Prague, Apolinarska 18, 128 51 Prague, Czech Republic; 3 Department of Clinical Biochemistry, Institute of Endocrinology, Narodni 8, 116 94 Prague, Czech Republic

**Keywords:** COVID-19, vaccination, infection, safety, woman fertility, AMH, antral follicle count, steroids

## Abstract

The fast-track process to approve vaccines against COVID-19 has raised questions about their safety, especially in relation to fertility. Over the last 2 years, studies have appeared monitoring female fertility, especially from assisted reproduction centers or in animal experiments. However, studies monitoring healthy populations are still limited. The aim of our study was to monitor the relevant parameters of female fertility (sex and other steroids, LH, FSH, SHBG, Antimüllerian hormone and antral follicle count) before and then 2–4 months after the third dose of vaccination against COVID-19 in a group of 25 healthy fertile woman. In addition, anti-SARS-CoV-2 and anti-SARS-CoV-2S antibodies were determined. We did not observe significant changes in the measured parameters before and after the third dose of vaccination. By comparing levels of the analytes with antibodies indicating a prior COVID-19 infection, we found that women who had experienced the disease had statistically lower levels of estrone, estradiol, SHBG and 5α-dihydroprogesterone, and conversely, higher levels of androgen active dehydroepiandrosterone and dihydrotestosterone. Our results confirm that vaccination does not affect female fertility, and that what fertile women should be worried about is not vaccination, but rather COVID-19 infection itself.

## 1. Introduction

The rapidly spreading COVID-19 pandemic accelerated the development of vaccines against this disease. The fast-track approval of vaccines has raised many questions about their safety in some parts of the population, and consequently there has been increased reluctance in some people to be vaccinated against the disease. One of the most widespread concerns among the least vaccinated group of people, i.e., women and men aged between 25 and 34 years, is concern for their reproductive health (*ockovani.opendatalab.cz*). This concern is reinforced through the spread of misinformation, particularly on social media platforms that reach young people most. Uncertainty is often fueled by distrust of vaccinations in general among the public, and by a lack of clarity and consistency in communication from health professionals. A study by American authors published in November 2021 shows that the vast majority of women planning a pregnancy have not been vaccinated against COVID-19 [1].

Official statements by the World Health Organization and the US Centers for Disease Control clearly declare that there is no evidence for concern about the negative effects of vaccination on fertility in women and men, and that vaccination does not cause infertility and cannot affect fertility treatments. On the contrary, vaccination is also appropriate for those planning or currently seeking pregnancy [2,3,4,5,6]. However, there are very few studies looking at associations between COVID-19 vaccination and fertility in women. This topic has been studied much more intensively in men [7,8,9,10], in spite of the fact that women are generally more concerned about problems with fertility.

Published data [11,12] show a declining efficacy of vaccination against COVID-19 over time. Receiving early booster doses can significantly influence this trend and provide stronger protection against COVID-19 [13]. National medical authorities in the vast majority of countries have issued recommendations for the administration of a third dose of a COVID-19 vaccine starting 6 months after the second dose, following recommendations by the European Medicines Agency (EMA) and the US Food and Drug Administration (FDA). The results of clinical studies have confirmed the high immunogenicity and good safety profile of the third dose [14].

The receptor for SARS-CoV-2 entry into human cells is Angiotensin Converting Enzyme 2 (ACE2), a major component of the renin-angiotensin system. Once the virus infects and enters the host cell, this system is disrupted and ACE2 expression in the host cell is reduced, leading to an increased pro-inflammatory response. The angiotensin and ACE2 system also regulate basic functions of the female and male reproductive systems. In women, this includes the genesis of follicles, oocyte maturation, ovulation, endometrial regeneration, and steroid secretion. ACE2 expression has also been detected in the ovary, oocytes, follicular fluid, cumulus cells, uterus, vagina, fallopian tube, cervix, endometrium, endometrial epithelial and stromal cells and myometrium [15,16,17]. As SARS-CoV-2 enters the cell via ACE2, cells/tissues of the reproductive system are also potentially vulnerable to this virus and their function could theoretically be affected [18]. The authors Jing et al. [15] even published in May 2020 that SARS-CoV-2 infection can impair reproductive function in women and cause sterility or infertility, but the publication was based on theoretical findings only and did not contain any experimental data. When scientific information of this kind reaches the public, instead of leading to fears of infection and a subsequent willingness to be vaccinated, it often provides a basis for misinformation about COVID-19 vaccines.

In the attachment to the host ACE2 receptor, transmembrane serine protease type 2 (TMPRSS2) is necessary, enabling the membrane fusion and subsequent entry of the SARS-CoV-2 virus. This is a cell surface protein primarily expressed by endothelial cells of the respiratory and digestive tract. A higher co-expression of TMPRSS2 and ACE2 has been reported in pneumocytes of males compared to females. This may also play role in the gender pattern of COVID-19 infection [19]. ACE2 receptor expression has been identified in the ovaries, oocytes, uterus, placenta, seminiferous tubules, Leydig cells, Sertoli cells, spermatozoa and adrenal glands, and TMPRSS2 has been identified in the epididymis, prostate glands, seminal vesicles and adrenal glands [15,20,21,22]. The TMPRSS2 gene also plays a role in the development and progression of prostate cancer [23]. It has also been reported that prostate cancer patients receiving androgen deprivation therapy had a significantly lower risk of SARS-CoV-2 infection compared to patients without this therapy [24], which highlights the role of steroids in this disease. In primates, the highest expression of ACE2 was found in the antral follicles [22], which means that oocytes are most vulnerable just before ovulation during the physiological cycle. Wang et al., however, reported that ovarian function and ovarian reserves during ART cycles are not impacted by SARS-CoV-2 infection [25]. SARS-CoV-2 infection was reported to have possible effects on menstrual cycle changes, ovarian tissue damage, decreases in ovarian function and oocyte quality [21,26,27]. The adrenal glands also seem to be affected by this virus; adrenal lesions, necrosis, cortical lipid degeneration, hemorrhage and unspecific local adrenitis have been reported after infection [28].

The aim of the study was to compare baseline parameters of female fertility, the steroid hormone spectrum and anti-SARS-CoV-2 antibodies in 25 women of childbearing age before and after the third dose of COVID-19 vaccination. The objectives included in particular: (1) a comparison of steroid hormone levels before and after vaccination, with a specific focus on estrogens and other sex hormones. (2) Comparisons of antral follicle count (AFC), Antimüllerian hormone (AMH), luteinizing hormone (LH), and follicle stimulating hormone (FSH) serum levels before and after vaccination. (3) An evaluation of the effect of vaccination on fertility-related changes and menstrual cycle changes. (4) A comparison of all measured parameters in women who have and who have not experienced COVID-19 disease. To the best of our knowledge, this is the first study monitoring relevant parameters of women fertility in relation to the third dose of COVID-19 vaccination.

## 2. Results

Overall, 88% of women received the Pfizer/BioNTech COVID-19 vaccine for their first and second doses, and 12% received the Moderna vaccine. In total, 24% of women had been infected with SARS-CoV-2 before the first vaccination. All women vaccinated with Moderna the first and second time received their third dose from the same manufacturer. Almost one-third of women initially vaccinated with the Pfizer/BioNTech vaccine decided to receive their third dose from Moderna.

After the first dose of either vaccine, 28% of women reported fatigue, 56% injection site pain, and 32% had no reaction to the vaccination. After the second dose, 64% reported fatigue, 45% injection site pain and elevated temperature, 20% headache, and 32% had no reaction. After the third dose, 48% reported fatigue, 60% injection site pain, 12% elevated temperature, 16% headache, and 20% had no reaction. These data may indicate that with the increased number of vaccine doses may also increase the overall reaction.

Concerning menstrual cycle changes, after the first and second vaccine doses, 92% of women did not observe changes in their cycle. After the third dose, the percentage of women reporting no cycle changes decreased to 64%, while 20% of woman reported cycle prolongation, 8% cycle shortening and 4% bleeding out of cycle. Similarly, to overall reactions, menstrual cycle changes occurred more frequently with increased doses of the COVID-19 vaccines. The complete data are summarized in Table 1.

Testing for the presence of anti-SARS-CoV-2 antibodies in the serum of woman from the first sampling (before the 3rd vaccine dose) showed that 24% of samples (6 woman) were reactive (>1 COI). These were the same women who reported having had a COVID-19 infection before the 1st dose of vaccine in the questionnaire. The results from the second sampling (2–4 months after the 3rd dose) showed that 68% of samples were reactive (indicating a COVID-19 infection). However, according to the questionnaire only 44% (11 woman) had overcome COVID-19 infections (six before the study and six during the study, but one woman had a reinfection). These results indicate that 24% of woman experienced a silent COVID-19 infection during the course of the study.

Concerning anti-SARS-CoV-2S antibodies monitoring the efficacy of vaccination, all samples before the 3rd dose of vaccination were reactive (higher than 0.8 U/mL), and 92% of samples exceeded the upper limit of detection (250 U/mL). After the 3rd dose of vaccination, all samples were reactive, and again 92% of samples exceeded the upper limit of detection.

We quantified plasma concentrations of 24 steroids covering all common as well as some less common biosynthetic pathways. Together with serum levels of LH, FSH, SHBG, AMH and AFC, this allowed us to assess potential disruptions to steroid hormone and fertility parameters as a result of COVID-19 vaccinations. Using the Wilcoxon test, we did not find significant changes in any of the analytes of interest in the samples collected before the third dose of vaccination and 2–4 months after. The levels of steroids, LH, FSH, SHBG, AMF and AFC in plasma/serum are given in Table 2.

**Figure 1:**
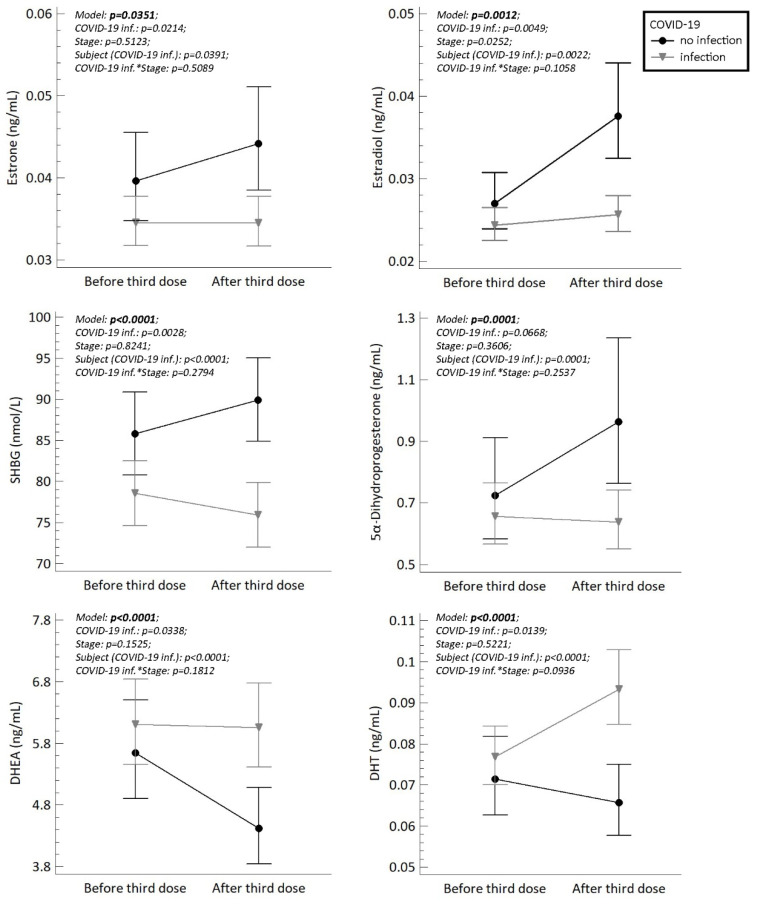
The plasma/serum levels of estrone, estradiol, SHBG (serum), 5α-dihydroprogesterone, DHEA and DHT in fertile women (n = 25) who were reactive (indicating a prior COVID-19 infection) and non-reactive (no infection) to anti-SARS-CoV-2 antibodies before and after the third dose of COVID-19 vaccination. The symbols (circles and triangles) with error bars represent retransformed means with 95% confidence intervals computed using the least significant difference multiple comparisons (*p* < 0.05). Confidence intervals that do not overlap denote significant differences between the respective subgroups of means. Statistical significances (*p*-values) are shown in each graph. Stage means status before or after third dose of vaccination. COVID-19 inf.*Stage represents the interaction between these variables.

Finally, we compared the steroid, LH, FSH, SHBG, AMH levels and AFC counts between woman who had been infected with the SARS-CoV-2 virus (reactive anti-SARS-CoV-2 antibodies, COI > 1) and woman who were not infected before or during the study. Because we cannot exclude possible silent COVID-19 reinfections in woman with positive anti-SARS-CoV-2 antibodies before the study, we classified all women with anti-SARS-CoV-2 antibodies COI > 1 as reactive and all woman with anti-SARS-CoV-2 antibodies COI ˂ 1 as non-reactive. The ANOVA model showed that women who had experienced a COVID-19 infection had significantly higher plasma levels of DHT and DHEA, and significantly lower levels of 5α-dihydroprogesterone, estrone, estradiol and SHBG at the time of the second sampling (after 3rd dose of COVID-19 vaccine). The results are summarized in Figure 1.

## 3. Discussion

Since the beginning of global effort to provide vaccinations against COVID-19, there has been a certain degree of fear associated with effects on fertility. The first study to evaluate the involvement of the ovaries in the immune response to COVID-19 disease or vaccination was a study from Israel by Bentov et al. [12] published in July 2021. The authors measured selected parameters of female fertility (AFC, AMH, LH, FSH, estradiol, and progesterone) from the serum and follicular fluid of patients obtained during oocyte retrieval as part of artificial insemination. While this was not a representative group of healthy women, it was the first study evaluating relevant fertility parameters. The group consisted of a total of 32 patients (9 vaccinated without having had COVID-19, 9 vaccinated after a COVID-19 infection, and 14 unvaccinated and without a COVID-19 infection). No differences were found between the groups. Another Israeli study looking at possible changes in fertility parameters was published in October 2021 [29]. Those authors observed changes in fertility parameters in women who underwent ovarian stimulation for artificial insemination before and after vaccination against COVID-19.

In May 2021, a group of American authors published a study on rats injected with the Pfizer/BioNTech vaccine [30]. After vaccination, no effects on mating, fertility, or the size or structure of the ovaries or uterus were observed. A British study published in July 2021 [31] monitored the effect of the AstraZeneca vaccine on fertility and reproductive function in CD-1 mice during the embryofetal developmental phase as well as postnatally. They also measured immune responses following the vaccination of females, including the response of fetuses and newborn pups. Each experiment was performed on 25 subjects and 25 controls. The authors compared common physiological parameters at gestation and in offspring, as well as mating ability, fertility, gestation length and uterine weight at gestation. The authors found no significant differences in any of the parameters studied in relation to COVID-19 vaccination.

The first study investigating AMH levels in healthy young woman before and after COVID-19 vaccination was published at the end of 2021, covering 129 reproductive age women vaccinated with two doses of the Pfizer/BioNTech vaccine [32]. Blood samples were collected before and 3 months after the first vaccine administration, and each woman served as her own control. Together with AMH monitoring, vaccination efficiency was also monitored using SARS-CoV-2 IgG antibodies. No significant differences in AMH levels were observed before and after vaccination, and no associations were found between antibody levels and AMH levels.

Our study was initiated in November 2021, which was the time when the third doses of vaccines started to be given in the Czech Republic. We decided to take advantage of this situation and monitor the effects of repeated vaccine doses, since no study had yet dealt with the third dose of vaccination. In accordance with previously published studies monitoring the first and second COVID-19 vaccines effects, we did not find any significant changes in the abovementioned fertility parameters or steroids before and then 2–4 months after the third dose of vaccination. 

With the ongoing pandemic, studies evaluating the effect of the disease itself on female fertility started to appear. A study from 2021 monitoring artificial reproductive technology (ART) cycles found no differences in ovarian reserve (measured using AMH over the course of one year) between woman who had recovered from a SARS-CoV-2 infection and non-infected woman [33]. At the beginning of 2022, a systematic literature review reported that no SARS-CoV-2 RNA was detected in the oocytes/follicular fluid of infected women, and no differences with regard to pregnancy rates of the percentage of healthy children were found between persons who recovered from the disease, vaccinated persons and control subjects. Vaccination also had no impact on live birth rates after ART. However, there is growing evidence that severe SARS-CoV-2 infection has a negative impact on male fertility, and there is an increased risk of complications among pregnant women with the infection [9]. The current data clearly show the benefits of vaccination during pregnancy compared to experiencing an infection [34]. Otherwise, IgM and IgG transmission from mother to fetus and testing for this have also been investigated [35,36]. Another study from an in vitro fertilization (IVF) center published in 2022 compared six women vaccinated against COVID-19, five women recovered from COVID-19, and nine healthy women from before the coronavirus pandemic. At the time of oocyte retrieval, follicular fluids were collected, estradiol, progesterone, FSH, AMH, AFC and selected cytokines were determined. Though the sample size was small, no significant differences were observed between the groups [37]. Another study performed in an IVF center examined woman undergoing IVF/ICSI (intracytoplasmic sperm injection) cycles. Estradiol and progesterone in blood and follicular fluid and AFC were monitored in 59 women (37 vaccinated, 22 not vaccinated). They were further divided according to the presence of serum antibodies to three groups: (1) positive anti-S (anti-spike levels) indicating COVID-19 vaccination, (2) positive anti-N (anti-nucleotide levels) indicating a COVID-19 infection, and (3) negative for both. No statistical differences in any of analytes were observed between the groups [38]. 

In our study, we measured a wide spectrum of steroids and fertility markers. Unlike the previous study mentioned above [37], we found significantly different levels of estradiol between woman who had and had not experienced a COVID-19 infection. These contrasting results may be due to differences in the study subjects, since our study was performed on a general fertile women population, not only within an IVF center, and all woman were in the course of being vaccinated. In line with the lower plasma estradiol levels in woman who had experienced COVID-19, we also found lower levels of estrone and SHBG, which is a key transporter of estrogens.

Estrogens are important regulators of female fertility, mainly controlling functions of the reproductive system, but they also affect most tissues and organs in the female body. They are predominately produced by gonads and the placenta, but also in many other tissues like breasts, bone, skin, adipose tissue, vasculature and also in the brain [39]. In fertile woman sex steroids are synthesized cyclically, with estradiol levels rising until ovulation and then decreasing to basal levels. They are necessary for the proper functioning of MC, which is the key foundation of female fertility [40]. SARS-CoV-2 is known to interfere with ACE2, and studies have shown that there are also associations with changes in steroid secretion, primarily in estrogens [15,41]. The protective effects of estrogens on the cardiovascular and bone systems of premenopausal women are well known. Estradiol and progesterone were found to down-regulate ACE2 expression, and there is some evidence that both steroids could exert a protective effect in women through direct antiviral and immune-mediated mechanisms [42]. The published data suggest that in the case of COVID-19, women have a milder disease course than men and also die less frequently from the disease [43]. The administration of estrogenically active substances in experimental animals infected by COVID-19 was found to reduce the presence of ACE2 in the airways. These substances also have the potential to reduce hyperimmune responses [44]. Our data showing changes in estrogen levels are in accordance with the proposed mechanism of SARS-CoV-2 interfering with ACE2 under the control of estrogens. In addition, our data also suggest that there may also be a bidirectional mechanism of action. 

Progesterone is also an important female steroid. It is a major gonadal hormone synthesized in the corpus luteum of the ovaries and also by the placenta during pregnancy [45,46,47]. Progesterone has a relatively short half-life in the body–only about 5 min. Approximately 50% is metabolized to 5α-dihydroprogesterone in the corpus luteum, 35% is metabolized in the liver to 3β-dihydroprogesterone, and 10% is metabolized to 20α-dihydroprogesterone [48]. 5α-dihydroprogesterone is the main progesterone metabolite, from which are formed neuroactive pregnane steroids, including allopregnanolone [49,50,51]. Lower plasma levels of 5α-dihydroprogesterone in woman infected with SARS-CoV-2 may also indicate the bidirectional influence of the SARS-CoV-2 virus to ACE2 and steroid expression. 

Since infection and vaccination in the context of COVID-19 has been studied more in men than in women, there are now much published data showing that androgens have an adverse effect on the course of SARS-CoV-2 infection [20,27,52]. A milder course of infection has been observed in men receiving androgen deprivation therapy than in the general male population. Dexamethasone has also been used successfully in the treatment of SARS-CoV-2 [53]. In our study, elevated levels of the androgen precursor DHEA and the main active androgen DHT were observed in the second samples (after the third vaccine dose) of woman who had been infected with the SARS-CoV-2 virus. This indicates that there may be complex interactions between SARS-CoV-2 infections, the entrance of the virus into the human cell, and the influence of steroidogenesis towards higher androgen and lower estrogen levels.

Most women of childbearing age are naturally interested in possible influences of COVID-19 infection and vaccination to their MC. While changes in the MC are not listed as one of the side effects of COVID-19 vaccination, there have been many reports from gynecological outpatient clinics where women have experienced MC disturbances after vaccination. These seem to mainly be early reactions to vaccination that return to normal over time, and are thought to be a general immune system response to the vaccination itself, not a reaction against specific components of the COVID-19 vaccines [54]. Changes to the MC have also been observed for other vaccines, e.g., for human papillomavirus [55]. The MC is sensitive to endogenous and exogenous factors that activate the immune system, including infections and lifestyle changes. Mechanisms linking immune activation to changes in the MC include effects of the immune system on hormones controlling the MC and effects mediated by immune cells present in the uterine lining that participate in the cyclic accumulation and breakdown [56]. It has been observed that SARS-CoV-2 infection, COVID-19 vaccination as well as stress associated with the COVID-19 pandemic can cause cycle changes [41]. The most common are irregular menstruation (60%), increased premenstrual syndromes (45%) and less frequent menstruation (35%) [57].

On the basis of our results, the majority of women experience no cycle changes after the first the two doses of the mRNA vaccines, but after the third dose more than one-third of woman report changes in their MC. This highlights the importance of MC monitoring in relation to COVID-19 vaccination as well as infection. Ambulant gynecologists in particular should be attuned to this issue.

We are aware of the limitations of our study due to the small sample number and the absence of healthy unvaccinated controls. It would be ideal to evaluate all analytes of interest in women who have not been vaccinated and have or have not been infected with the SARS-CoV-2 virus. After more than 2.5 years of the COVID-19 pandemic, however, it will be challenging to find fertile unvaccinated woman who have not yet been exposed to this virus.

## 4. Materials and Methods

### 4.1. Chemicals and Reagents

The steroids cortisol, cortisone and dehydroepiandrosterone (DHEA) were from Koch-Light Laboratories Ltd. (Suffolk, Great Britain); 7α-hydroxy-DHEA (7α-OH-DHEA), 7β-hydroxy-DHEA (7β-OH-DHEA), 7-oxo-DHEA, testosterone, androstenedione, pregnenolone, 17-hydroxy-pregnenolone (17-OH-Pregnenolone, corticosterone, 17-hydroxy-progesterone (17-OH-Progesterone), estrone, 17β-estradiol, estriol and deuterated standards of DHEA (D3-DHEA), androstenedione (D7-androstenedione), pregnenolone (D4-pregnenolone), 17-OH-pregnenolone (D3-17-OH-preg), dihydrotestosterone (DHT) (D3-DHT), D9-progesterone, D8-17-OH-progesterone, D8-corticosterone, D8-5α-dihydroprogesterone and deuterated standards of estrone (D4-estrone) and estriol (D2-estriol) were from Steraloids (Newport, RI, USA); 11β-hydroxy-androstenedione (11β-OH-androstenedione), 11-deoxycortisol (11-DOF), 21-deoxycortisol (21-DOF), 11-deoxycorticosterone (11-DOC), DHT, 5α-dihydroprogesterone, D3-estradiol (D3-E2), and D5-11DOF were from Sigma-Aldrich (St. Louis, MO, USA), 11-keto-testosterone was from Chromservis (Prague, Czech Republic), and progesterone was from Merck (Darmstadt, Germany). D8-21DOF, D4-11β-OH-androstenedione, D3-11keto-testosterone were from Cambridge Isotope Laboratories, Inc. (Tewksbury, MA, USA); D4-cortisol was obtained from CDN isotopes (Ponte-Claire, QC, Canada), and D1-testosterone was synthesized by Sci-Tech (Prague, Czech Republic). A deuterated standard of 17β-estradiol (D3-estradiol) was purchased from Sigma-Aldrich (St. Louis, MO, USA), as were 99,9% tert-butyl methyl ether, acetone, sodium bicarbonate, dansyl chloride and D7-cortisone. LC-MS grade methanol (Chromasolv, tested for UHPLC-MS, ≥99.9%,) and water (Chromasolv, ultra-tested for UHPLC-MS) were from Riedel-de Haën (Charlotte, NC, USA), ammonium fluoride (NH4F, an eluent additive for LC–MS, ≥98.0%) was from VWR International (Stribrna Skalice, Czech Republic), and physiological solution (0.9% sodium chloride) was from Ardeapharma, a.s. (Sevetin, Czech Republic).

### 4.2. Study Group

Thirty-six healthy fertile women (aged 20–43) were enrolled in the study between November 2021 and March 2022. All women were of Czech nationality and middle-European origin. The exclusion criteria were the use of drugs affecting steroidogenesis, including various types of hormonal contraception, pregnancy and lactation. The study was performed in accordance with the Declaration of Helsinki (2000) of the World Medical Association. The protocol was approved by the Ethics Committee of the General University Hospital in Prague (protocol number 101/21) and by the Ethics committee of the Institute of Endocrinology in Prague. Informed written consent with the use of biological materials for research reasons was obtained from all subjects participating in the project. All women were introduced to the project and allowed to study detailed project information, and were given the opportunity to ask any questions.

Samples of venous blood were collected before and then 2–4 months after the third dose of vaccination against COVID-19 (Pfizer/BioNTech or Moderna). The Pfizer/BioNTech and Moderna vaccines are the most frequently applied vaccines in the Czech Republic—both are novel mRNA vaccines applied in the two-dose basic scheme. For this reason, only participants vaccinated with these two vaccines were included in the study. Blood was taken from the cubital vein between 8:00 and 10:00 o’clock within the 1st–3rd day of the menstrual cycle (MC). Eight milliliters of blood was collected in collection tubes with separation gel (serum), and 8 mL of blood was collected in collection tubes with K2EDTA (plasma), immediately centrifuged (5 min, 2000 g, 4 °C), and the serum/plasma was then transferred to glass tubes and stored at −20 °C until analysis. The plasma was used for liquid chromatography-tandem mass spectrometry (LC-MS/MS) analyses, and the serum for immunochemical analyses.

During the follicular phase of the MC (until the 12th day), vaginal ultrasonography was performed to determine the antral follicle count (AFC). Ultrasound was performed before and then 2–4 months after the third dose of vaccination against COVID-19. At the same time, all women filled in a questionnaire, with results summarized in Table 3. The questionnaire asked about their age, weight, height, BMI, age of first menstruation, average cycle length, the COVID-19 vaccine manufacturer, side effects and MC changes after the first, second and third doses, COVID-19 infection and disease course, the use of hormonal contraception in the past, past pregnancies past (deliveries, abortions, or miscarriages), problems conceiving, smoking, alcohol abuse, medication, dietary habits and physical activity. Twenty-five women completed the entire study. Three women became pregnant during the study (two of them after the third dose of vaccination, and one woman decided not to get vaccinated because of early pregnancy), and eight women decided to not receive a third vaccine dose because of COVID-19 and other respiratory infections.

### 4.3. Determination of Steroids

The plasma levels of unconjugated steroids were measured by means of two LC-MS/MS methods described elsewhere [58,59].

The first method enables the quantification of levels of unconjugated estrogens in human plasma [58]. In brief, the sample preparation was as follows: 500 µL of plasma was spiked with an internal standard solution and diluted with 500 µL of physiological solution. Samples were extracted with 2 mL of tert-butyl methyl ether for 1 min; the aqueous phase was frozen in solid carbon dioxide, while the organic phase was transferred to a glass tube and the solvent was evaporated until dryness. In the same way, an eight-point calibration was performed. The samples were further derivatized using dansyl chloride. Briefly, 50 μL of 100 mM sodium bicarbonate buffer and 50 μL of dansyl chloride in acetone (1 mg/mL) were added to dry residues, vortexed, incubated (60 °C; 5 min), cooled to laboratory temperature and evaporated. The dry residues were reconstituted in 300 μL of methanol. The sample was finally diluted 1:1 with a 10 mM aqueous solution of ammonium formate, and 50 μL was injected in the LC-MS/MS for analysis. The method was performed using an API 3200 (Sciex, Concord, NC, USA) triple stage quadrupole mass spectrometer with electrospray ionization (ESI) connected to an ultra-high performance liquid chromatograph (UPLC) Eksigent ultraLC 110 system (Redwood City, CA, USA). Chromatographic separation of estrogens was carried out on a Kinetex C18 (100 × 3.0 mm, 1.7 µm) column (Phenomenex, Torrance, CA, USA) with a corresponding security guard at a flow rate 0.4 mL/min at 50 °C. Further analytical and validation details are reported in Vitku et al., 2015 [60] and Kolatorova Sosvorova et al., 2017 [58].

The second method was used to quantify the levels of the immunoactive steroids DHEA, 7α-OH-DHEA, 7β-OH-DHEA, 7-oxo-DHEA; the androgens testosterone, DHT, 11-ketotestrosterone, 11-OH testosterone, androstenedione, 11β-OH-androstenedione; and the C-21 steroids cortisol, 21-DOF, 11-DOF, cortisone, corticosterone, 11-DOC, pregnenolone, 17-OH-pregnenolone, 17-OH-progesterone, 5α-dihydroprogestrone and progesterone. Briefly, 500 µL of plasma was spiked with an internal standard solution and diluted with 500 µL of physiological solution. Samples were extracted with 2 mL of tert-butyl methyl ether for 1 min; the water phase was frozen in solid carbon dioxide, while the organic phase was transferred to a glass tube and the solvent evaporated. In the same way, an eight-point calibration was performed. Samples were reconstituted with 100 µL of 50% methanol, transferred into an Eppendorf tube and centrifuged at 15,000× *g* for 5 min. The clarified supernatant was transferred to an LC vial insert for LC-MS/MS analysis. The measurements were performed with an Exion LC AD system connected to a Sciex QTRAP 6500+ mass spectrometer (Sciex, Concord, NC, USA). Chromatographic separation was carried out using a Kinetex C18 column (100 mm × 3.0 mm, 2.6 µm) and SecurityGuard ULTRA cartridge system (UHPLC C18 for a 3 mm ID column), both purchased from Phenomenex (Torrance, CA, USA), at a flow rate of 0.6 mL/min at 40 °C. All analytical details were reported in Simkova et al., 2022 [59].

### 4.4. Determination of LH, FSH, SHBG, AMH, Anti-SARS-CoV-2 and Anti-SARS-CoV-2S

Electro-chemiluminescence immunoassays (ECLIA), performed on Cobas^®^ 6000, Roche Diagnostics International Ltd. (Rotkreuz, Switzerland), were used to measure LH, FSH, sex hormone binding globulin (SHBG), AMH, anti-SARS-CoV-2 and anti-SARS-CoV-2S in human serum. The individual method performance characteristics are summarized in Table 4.

The majority of serologic assays for the determination of anti-SARS-CoV-2 antibodies are qualitative and use either full-length or truncated versions of the nucleocapsid (N) or spike (S) protein as the target for antibody detection. One assay of this type (Elecsys^®^Anti-SARS-CoV-2) was used to evaluate the presence of prior SARS-CoV-2 infections. Roche has also developed a quantitative serologic assay that measures antibodies including IgG against the receptor binding domain of the S-protein, the target of vaccines, and thus may aid in the characterization of the immune response to vaccines. This assay (Elecsys^®^Anti-SARS-CoV-2S) was used to evaluate the vaccine efficacy.

### 4.5. Statistical Analysis

Based on conventional practice, the data that were below the limit of detection (LOD) were replaced by LOD/√2 [61]. Due to the non-Gaussion data distribution, a non-parametric analogue of the paired *t*-test–the Wilcoxon- test–was used to explore differences in steroid, LH, FSH, SHBG, AMH, anti-SARS-CoV-2 and anti-SARS-CoV-2S antibody levels and AFC counts in plasma/serum before and after the 3rd dose of the COVID-19 vaccine.

The relationships between the abovementioned dependent variables and the presence or absence of COVID-19 infection (based on the positivity/negativity of anti-SARS-CoV-2 antibodies) were evaluated using a repeated measures ANOVA model consisting of the following factors: stage (before and after the 3rd dose), subject (explaining inter-individual variability) and COVID-19 infection (presence/absence). The ANOVA model was followed by least significant difference (LSD) multiple comparisons. To eliminate a skewed data distribution and heteroscedasticity, the original data were transformed by Box–Cox transformation to attain a Gaussian distribution and constant variance before further processing.

The statistical software Statgraphics Centurion XVI from Statpoint Inc. (Warrenton, VA, USA) was used for data comparisons.

## 5. Conclusions

The results of our study performed in the Czech Republic confirm previously published findings that vaccination does not affect female fertility. Our data show that the third dose of the COVID-19 vaccine causes a higher rate of negative side effects and is more likely to cause changes in the MC. Despite this, we did not observe any statistically significant changes in steroid levels including female sex hormones, other laboratory markers of female fertility (LH, FSH, AMH) and AFC before and then 2–4 months after the third dose of vaccination. 

We also compared the levels of all analytes in women who had and had not experienced COVID-19 infections. Women who had been infected with the disease had statistically lower levels of estrone, estradiol, SHBG and 5α-dihydroprogesterone, and, conversely, higher levels of DHEA and DHT. These data indicate a bidirectional influence of SARS-CoV-2 and ACE2 on steroidogenesis. Our results also support the conclusion that fertile women should not be worried about vaccination, but rather about COVID-19 infection itself.

## Figures and Tables

**Table 1:** The overall reactions and menstrual cycle changes after the first, second and third doses of COVID-19 vaccines. MC—menstrual cycle.

Characteristics	n (%)	Characteristics	n (%)
*COVID-19 vaccine manufacturer-1st and 2nd dose*		*COVID-19 infection before vaccination*	
Pfizer/BioNTech	22 (88)	Yes-all mild disease course	6 (24)
Moderna	3 (12)	No	19 (76)
*Reaction after the 1st dose of vaccine*		*MC changes after the 1st dose of vaccine*	
Fatigue	7 (28)	MC shortening	1 (4)
Injection site pain	14 (56)	MC prolongation	1 (4)
Elevated temperature	1 (4)	Bleeding out of the cycle	0 (0)
Headache	2 (8)	Headache	0 (0)
Chills	0 (0)	Premenstrual syndrome	0 (0)
Vertigo	0 (0)	Ovulation pain	0 (0)
None	8 (32)	None	23 (92)
*Reaction after the 2nd dose of vaccine*		*MC changes after the 2nd dose of vaccine*	
Fatigue	16 (64)	MC shortening	1 (4)
Injection site pain	12 (48)	MC prolongation	1 (4)
Elevated temperature	3 (48)	Bleeding out of the cycle	0 (0)
Headache	5 (20)	Headache	0 (0)
Chills	1 (4)	Premenstrual syndrome	0 (0)
Vertigo	1 (4)	Ovulation pain	0 (0)
None	8 (32)	None	23 (92)
*COVID-19 vaccine manufacturer-3rd dose*		*COVID-19 infection during the study*	
Pfizer/BioNTech	15 (60)	Yes-all mild disease course	6 (24) (4 Omicron)
Moderna	10 (40)	No	19 (76)
*Reaction after the 3rd dose of vaccine*		*MC after the 3rd dose of vaccine*	
Fatigue	12 (48)	MC shortening	2 (8)
Injection site pain	15 (60)	MC prolongation	5 (20)
Elevated temperature	3 (12)	Bleeding out of the cycle	1 (4)
Headache	4 (16)	Headache	0 (0)
Chills	0 (0)	Premenstrual syndrome	0 (0)
Vertigo	0 (0)	Ovulation pain	0 (0)
None	5 (20)	None	16 (64)

**Table 2:** The levels of steroids, LH, FSH, SHBG, AMH and AFC in plasma/serum of women before and 2–4 months after 3rd dose of COVID-19 vaccination. Medians with lower and upper quartiles (in parentheses) are given. *p*-value indicates statistical significance; no significant differences were found for any of the analytes.

Analyte (Steroids in ng/mL)	Before the 3rd Dose of COVID-19 Vaccine	After the 3rd Dose of COVID-19 Vaccine	*p*-Value
Estrone	0.035 (0.029, 0.046)	0.0375 (0.031, 0.044)	0.424
Estradiol	0.024 (0.021, 0.032)	0.0299 (0.023, 0.040)	0.424
Estriol	0.003 (0.001, 0.005)	0.004 (0.002, 0.007)	0.23
Progesterone	0.097 (0.052, 0.146)	0.103 (0.063, 0.132)	0.838
17-OH-Progesterone	0.315 (0.283, 0.446)	0.396 (0.268, 0.556)	0.214
5α-Dihydroprogesterone	0.656 (0.346, 1.106)	0.696 (0.396, 1.611)	0.831
Pregnenolone	0.928 (0.755, 1.291)	0.97 (0.620, 1.708)	0.953
17-OH-Pregnenolone	1.322 (0.888, 2.320)	1.137 (0.522, 2.527)	0.838
DHEA	6.807 (4.071, 9.248)	5.114 (3.900, 9.404)	0.689
7α-OH-DHEA	0.252 (0.115, 0.376)	0.188 (0.115, 0.382)	0.359
7β-OH-DHEA	0.092 (0.067, 0.115)	0.079 (0.060, 0.132)	0.383
7-oxo-DHEA	0.032 (0.024, 0.055)	0.028 (0.024, 0.068)	0.264
Testosterone	0.224 (0.166, 0.263)	0.215 (0.176, 0.266)	0.368
DHT	0.078 (0.058, 0.096)	0.077 (0.054, 0.121)	0.689
Androstenedione	0.89 (0.646, 1.045)	0.793 (0.669, 1.219)	0.424
11β-OH-Androstenedione	1.21 (0.899, 1.702)	1.01 (0.738, 1.532)	0.23
11-OH-Testosterone	0.135 (0.112, 0.166)	0.127 (0.091, 0.174)	0.54
11-Keto-testosterone	0.307 (0.224, 0.395)	0.318 (0.208, 0.405)	0.525
Cortisol	144 (128, 153)	131 (114, 167)	0.424
Cortisone	29.9 (26, 34.8)	27.5 (23.0, 35.7)	0.935
Corticosterone	2.404 (1.440, 4.007)	2.038 (1.634, 3.646)	0.4
11-Deoxycortisol	0.339 (0.217, 0.451)	0.311 (0.190, 0.479)	0.567
21-Deoxycortisol	0.026 (0.017, 0.049)	0.029 (0.012, 0.055)	0.831
11-Deoxycorticosterone	0.035 (0.026, 0.053)	0.043 (0.025, 0.057)	0.831
LH (IU/L)	6.36 (4.80, 7.42)	6.11 (5.70, 7.50)	0.424
FSH (IU/L)	6.2 (5.8, 8.6)	6.49 (5.045, 8.660)	0.424
SHBG (nmol/L)	67.4 (49.7, 105.3)	70.17 (56.76, 97.50)	0.75
AMH (ng/mL)	3.25 (1.46, 5.09)	3.03 (1.68, 5.04)	0.689
AFC	23 (19.5, 28.75)	24 (21.50, 28.75)	0.19

**Table 3:** Characteristics of the study group (*n* = 25). MC—menstruation cycle.

Characteristics	n (%)	Characteristics	n (%)
*Age (years)*		*BMI*	
<25	6 (24)	<18.5	2 (8)
25–35	13 (52)	18.5–25	15 (60)
>35	6 (24)	>25	8 (32)
Mean (±STD)	30(6.8)	Mean (±STD)	24.1 (5.3)
*Average length of MC*		*First MC*	
<26	2 (8)	<11	2 (8)
26–30	17 (68)	11–13	18 (72)
>30	6 (24)	>13	5 (20)
Mean (±STD)	28.8 (2.8)	Mean (±STD)	12.5 (1.4)
*Smoking*		*Alcohol*	
Yes	2 (8)	Regularly	2 (8)
No	19 (76)	Occasionally	21 (84)
In the past	4 (16)	None	2 (8)
*Eating habits*		*Physical activity*	
Common	19 (76)	Common	16 (64)
Vegetarian/vegan	3 (12)	Fitness sport	7 (28)
Intermittent fasting	2 (8)	Competitive sport	2 (8)
Diets	1 (4)		
*Contraception in the past*		*Pregnancy in the past*	
Yes	15 (60)	Yes	4 (16)
No	10 (40)	−3 women have children	
		No	21 (84)

**Table 4:** Immunoassay method performance characteristics.

Method	Units	Measuring Range	Limit of Detection	Reference Range/ Data Interpretation
Elecsys^®^LH	IU/L	0.100–200	0.1	women in follicular phase: 2.4–12.6
Elecsys^®^FSH	IU/L	0.100–200	<0.100	women in follicular phase: 3.5–12.5
Elecsys^®^AMH	ng/mL	0.01–23	0.01	women 20–24 years: 1.22–11.7 women 25–29 years: 0.890–9.85 women 30–34 years: 0.576–8.13 women 35–39 years 0.147–7.49 women 40–44 years: 0.027–5.47
Elecsys^®^SHBG	nmol/L	0.350–200	0.35	43–95
Elecsys^®^Anti-SARS-CoV-2	COI	qualitative	99.5% of specificity	≥1.0 indicates positive
Elecsys^®^Anti-SARS-CoV-2 S	U/mL	0.40–250	0.35	≥0.8 indicates positive

## Data Availability

The data presented in this study are available on reasonable request from corresponding author.

## References

[1] Toth-Manikowski S.M., Swirsky E.S., Gandhi R., Piscitello G. (2021). COVID-19 vaccination hesitancy among health care workers, communication, and policy-making. Am. J. Infect. Control.

[2] Schaler L., Wingfield M. (2021). COVID-19 vaccine-can it affect fertility?. Ir. J. Med. Sci..

[3] Hillson K., Clemens S.C., Madhi S.A., Voysey M., Pollard A.J., Minassian A.M. (2021). Fertility rates and birth outcomes after ChAdOx1 nCoV-19 (AZD1222) vaccination. Lancet.

[4] (2021). Stanovisko České Lékařské Společnosti (ČLS) Jana Evangelisty Purkyně (JEP); České Gynekologické a Porodnické Společnosti ČLS JEP. Očkování Proti Onemocnění COVID-19 u Těhotných a Kojících Žen. https://koronavirus.mzcr.cz/wp-content/uploads/2021/06/Stanovisko-k-očkován%C3%AD-proti-onemocněn%C3%AD-covid-19-u-těhotných-a-koj%C3%ADc%C3%ADch-žen.pdf.

[5] Han A.R., Lee D., Kim S.K., Choo C.W., Park J.C., Lee J.R., Choi W.J., Jun J.H., Rhee J.H., Kim S.H. (2022). Effects and safety of COVID-19 vaccination on assisted reproductive technology and pregnancy: A comprehensive review and joint statements of the KSRM, the KSRI, and the KOSAR. Clin. Exp. Reprod. Med..

[6] (2022). Statement—COVID-19 Vaccination-Male and Female fertility, treatments to get pregnant, pregnancy. JBRA Assist. Reprod..

[7] Gonzalez D.C., Nassau D.E., Khodamoradi K., Ibrahim E., Blachman-Braun R., Ory J., Ramasamy R. (2021). Sperm Parameters before and after COVID-19 mRNA Vaccination. JAMA.

[8] Chen F., Zhu S., Dai Z., Hao L., Luan C., Guo Q., Meng C., Zhang Y. (2021). Effects of COVID-19 and mRNA vaccines on human fertility. Hum. Reprod..

[9] Braun A.S., Feil K., Reiser E., Weiss G., von Steuben T., Pinggera G.M., Kohn F.M., Toth B. (2022). Corona and Reproduction, or Why the Corona Vaccination Does Not Result in Infertility. Geburtshilfe Frauenheilkd.

[10] Mirza S.A., Sheikh A.A.E., Barbera M., Ijaz Z., Javaid M.A., Shekhar R., Pal S., Sheikh A.B. (2022). COVID-19 and the Endocrine System: A Review of the Current Information and Misinformation. Infect. Dis. Rep..

[11] Markert U.R., Szekeres-Bartho J., Schleussner E. (2021). Adverse effects on female fertility from vaccination against COVID-19 unlikely. J. Reprod. Immunol..

[12] Bentov Y., Beharier O., Moav-Zafrir A., Kabessa M., Godin M., Greenfield C.S., Ketzinel-Gilad M., Ash Broder E., Holzer H.E.G., Wolf D. (2021). Ovarian follicular function is not altered by SARS-CoV-2 infection or BNT162b2 mRNA COVID-19 vaccination. Hum. Reprod..

[13] Galanis P., Vraka I., Katsiroumpa A., Siskou O., Konstantakopoulou O., Katsoulas T., Mariolis-Sapsakos T., Kaitelidou D. (2022). First COVID-19 Booster Dose in the General Population: A Systematic Review and Meta-Analysis of Willingness and Its Predictors. Vaccines.

[14] Doporučení ČLS JEP (ČVS) (2021). ČLS JEP (ČSAKI) a ČLS JEP (SEM) k Přeočkování a Aplikaci Dodatečných (Třetích) Dávek Vakcíny Proti Onemocnění COVID-19. https://www.infekce.cz/zprava21-45.htm.

[15] Jing Y., Li R.-Q., Wang H.-R., Chen H.-R., Liu Y.-B., Yang G., Fei C. (2020). Potential influence of COVID-19/ACE2 on the female reproductive system. Mol. Hum. Reprod..

[16] Zupin L., Pascolo L., Zito G., Ricci G., Crovella S. (2020). SARS-CoV-2 and the next generations: Which impact on reproductive tissues?. J. Assist. Reprod. Genet..

[17] Morelli F., Meirelles L.E.F., de Souza M.V.F., Mari N.L., Mesquita C.S.S., Dartibale C.B., Damke G., Damke E., da Silva V.R.S., Souza R.P. (2021). COVID-19 Infection in the Human Reproductive Tract of Men and Nonpregnant Women. Am. J. Trop. Med. Hyg..

[18] Madjunkov M., Dviri M., Librach C. (2020). A comprehensive review of the impact of COVID-19 on human reproductive biology, assisted reproduction care and pregnancy: A Canadian perspective. J. Ovarian Res..

[19] Song H., Seddighzadeh B., Cooperberg M.R., Huang F.W. (2020). Expression of ACE2, the SARS-CoV-2 receptor, and TMPRSS2 in prostate epithelial cells. Eur. Urol..

[20] Dutta S., Sengupta P. (2020). SARS-CoV-2 infection, oxidative stress and male reproductive hormones: Can testicular-adrenal crosstalk be ruled-out?. J. Basic Clin. Physiol. Pharmacol..

[21] Lu M., Qiu L., Jia G., Guo R., Leng Q. (2020). Single-cell expression profiles of ACE2 and TMPRSS2 reveals potential vertical transmission and fetus infection of SARS-CoV-2. Aging.

[22] Stanley K.E., Thomas E., Leaver M., Wells D. (2020). Coronavirus disease-19 and fertility: Viral host entry protein expression in male and female reproductive tissues. Fertil. Steril..

[23] Mollica V., Rizzo A., Massari F. (2020). The pivotal role of TMPRSS2 in coronavirus disease 2019 and prostate cancer. Future Oncol..

[24] Montopoli M., Zumerle S., Vettor R., Rugge M., Zorzi M., Catapano C.V., Carbone G.M., Cavalli A., Pagano F., Ragazzi E. (2020). Androgen-deprivation therapies for prostate cancer and risk of infection by SARS-CoV-2: A population-based study (N = 4532). Ann. Oncol..

[25] Wang M., Yang Q., Ren X., Hu J., Li Z., Long R., Xi Q., Zhu L., Jin L. (2021). Investigating the impact of asymptomatic or mild SARS-CoV-2 infection on female fertility and in vitro fertilization outcomes: A retrospective cohort study. eClinicalMedicine.

[26] Singh B., Gornet M., Sims H., Kisanga E., Knight Z., Segars J. (2020). Severe Acute Respiratory Syndrome Coronavirus 2 (SARS-CoV-2) and its effect on gametogenesis and early pregnancy. Am. J. Reprod. Immunol..

[27] Knizatova N., Massanyi M., Roychoudhury S., Guha P., Greifova H., Tokarova K., Jambor T., Massanyi P., Lukac N. (2021). Is there impact of the SARS-CoV-2 pandemic on steroidogenesis and fertility?. Physiol. Res..

[28] Freire Santana M., Borba M.G.S., Baia-da-Silva D.C., Val F., Alexandre M.A.A., Brito-Sousa J.D., Melo G.C., Queiroga M.V.O., Leao Farias M.E., Camilo C.C. (2020). Case Report: Adrenal Pathology Findings in Severe COVID-19: An Autopsy Study. Am. J. Trop. Med. Hyg..

[29] Orvieto R., Noach-Hirsh M., Segev-Zahav A., Haas J., Nahum R., Aizer A. (2021). Does mRNA SARS-CoV-2 vaccine influence patients’ performance during IVF-ET cycle?. Reprod. Biol. Endocrinol..

[30] Bowman C.J., Bouressam M., Campion S.N., Cappon G.D., Catlin N.R., Cutler M.W., Diekmann J., Rohde C.M., Sellers R.S., Lindemann C. (2021). Lack of effects on female fertility and prenatal and postnatal offspring development in rats with BNT162b2, a mRNA-based COVID-19 vaccine. Reprod. Toxicol..

[31] Stebbings R., Maguire S., Armour G., Jones C., Goodman J., Maguire A.K., Tang C.M., Skellett V., Harris J. (2021). Developmental and reproductive safety of AZD1222 (ChAdOx1 nCoV-19) in mice. Reprod. Toxicol..

[32] Mohr-Sasson A., Haas J., Abuhasira S., Sivan M., Doitch Amdurski H., Dadon T., Blumenfeld S., Derazne E., Hemi R., Orvieto R. (2022). The effect of COVID-19 mRNA vaccine on serum anti-Mullerian hormone levels. Hum. Reprod..

[33] Kolanska K., Hours A., Jonquiere L., Mathieu d’Argent E., Dabi Y., Dupont C., Touboul C., Antoine J.M., Chabbert-Buffet N., Darai E. (2021). Mild COVID-19 infection does not alter the ovarian reserve in women treated with ART. Reprod. Biomed. Online.

[34] Cavaliere A.F., Zaami S., Pallottini M., Perelli F., Vidiri A., Marinelli E., Straface G., Signore F., Scambia G., Marchi L. (2021). Flu and Tdap Maternal Immunization Hesitancy in Times of COVID-19: An Italian Survey on Multiethnic Sample. Vaccines.

[35] Cavaliere A.F., Marchi L., Aquilini D., Brunelli T., Vasarri P.L. (2021). Passive immunity in newborn from SARS-CoV-2-infected mother. J. Med. Virol..

[36] Cavaliere A.F., Carabaneanu A.I., Perelli F., Matarrese D., Brunelli T., Casprini P., Vasarri P.L. (2022). Universal screening for SARS-CoV-2 in pregnant women admitted for delivery: How to manage antibody testing?. J. Mattern. Fetal Neonatal Med..

[37] Castiglione Morelli M.A., Iuliano A., Schettini S.C.A., Ferri A., Colucci P., Viggiani L., Matera I., Ostuni A. (2022). Are the Follicular Fluid Characteristics of Recovered Coronavirus Disease 2019 Patients Different from Those of Vaccinated Women Approaching in vitro Fertilization?. Front. Physiol..

[38] Odeh-Natour R., Shapira M., Estrada D., Freimann S., Tal Y., Atzmon Y., Bilgory A., Aslih N., Abu-Raya Y.S., Shalom-Paz E. (2022). Does mRNA SARS-CoV-2 vaccine in the follicular fluid impact follicle and oocyte performance in IVF treatments?. Am. J. Reprod. Immunol..

[39] Cui J., Shen Y., Li R. (2013). Estrogen synthesis and signaling pathways during aging: From periphery to brain. Trends Mol. Med..

[40] Findlay J.K., Liew S.H., Simpson E.R., Korach K.S. (2010). Estrogen signaling in the regulation of female reproductive functions. Handb. Exp. Pharmacol..

[41] Li K., Chen G., Hou H., Liao Q., Chen J., Bai H., Lee S., Wang C., Li H., Cheng L. (2021). Analysis of sex hormones and menstruation in COVID-19 women of child-bearing age. Reprod. Biomed. Online.

[42] Cattrini C., Bersanelli M., Latocca M.M., Conte B., Vallome G., Boccardo F. (2020). Sex Hormones and Hormone Therapy during COVID-19 Pandemic: Implications for Patients with Cancer. Cancers.

[43] Traish A.M. (2021). Sex steroids and COVID-19 mortality in women. Trends Endocrinol. Metab..

[44] Smetana K., Jakubek M., Drábek J. (2021). Chrání estrogeny před těžkým průběhem COVID-19?. Vesmír.

[45] Sundstrom-Poromaa I., Comasco E., Sumner R., Luders E. (2020). Progesterone-Friend or foe?. Front. Neuroendocrinol..

[46] Zhang Y., Nadeau M., Faucher F., Lescelleur O., Biron S., Daris M., Rheaume C., Luu-The V., Tchernof A. (2009). Progesterone metabolism in adipose cells. Mol. Cell. Endocrinol..

[47] Rossato M., Nogara A., Merico M., Ferlin A., Foresta C. (1999). Identification of functional binding sites for progesterone in rat Leydig cell plasma membrane. Steroids.

[48] Anderson G.D., Odegard P.S. (2004). Pharmacokinetics of estrogen and progesterone in chronic kidney disease. Adv. Chronic Kidney Dis..

[49] Kancheva R., Hill M., Cibula D., Vcelakova H., Kancheva L., Vrbikova J., Fait T., Parizek A., Starka L. (2007). Relationships of circulating pregnanolone isomers and their polar conjugates to the status of sex, menstrual cycle, and pregnancy. J. Endocrinol..

[50] Hill M., Cibula D., Havlikova H., Kancheva L., Fait T., Kancheva R., Parizek A., Starka L. (2007). Circulating levels of pregnanolone isomers during the third trimester of human pregnancy. J. Steroid Biochem. Mol. Biol..

[51] Hirst J.J., Kelleher M.A., Walker D.W., Palliser H.K. (2014). Neuroactive steroids in pregnancy: Key regulatory and protective roles in the foetal brain. J. Steroid Biochem. Mol. Biol..

[52] Duarte-Neto A.N., Monteiro R.A.A., da Silva L.F.F., Malheiros D., de Oliveira E.P., Theodoro-Filho J., Pinho J.R.R., Gomes-Gouvea M.S., Salles A.P.M., de Oliveira I.R.S. (2020). Pulmonary and systemic involvement in COVID-19 patients assessed with ultrasound-guided minimally invasive autopsy. Histopathology.

[53] Stárka L., Dušková M. (2021). Androgeny v nákaze koronavirem SARS-CoV-2. Diabetol. Metab. Endokrinol. Vyziv..

[54] Male V. (2021). Menstrual changes after COVID-19 vaccination. BMJ.

[55] Suzuki S., Hosono A. (2018). No association between HPV vaccine and reported post-vaccination symptoms in Japanese young women: Results of the Nagoya study. Papillomavirus Res..

[56] Monin L., Whettlock E.M., Male V. (2020). Immune responses in the human female reproductive tract. Immunology.

[57] Khan S.M., Shilen A., Heslin K.M., Ishimwe P., Allen A.M., Jacobs E.T., Farland L.V. (2022). SARS-CoV-2 infection and subsequent changes in the menstrual cycle among participants in the Arizona CoVHORT study. Am. J. Obstet. Gynecol..

[58] Kolatorova Sosvorova L., Chlupacova T., Vitku J., Vlk M., Heracek J., Starka L., Saman D., Simkova M., Hampl R. (2017). Determination of selected bisphenols, parabens and estrogens in human plasma using LC-MS/MS. Talanta.

[59] Simkova M., Kolatorova L., Drasar P., Vitku J. (2022). An LC-MS/MS method for the simultaneous quantification of 32 steroids in human plasma. J. Chromatogr. B Anal. Technol. Biomed. Life Sci..

[60] Vitku J., Chlupacova T., Sosvorova L., Hampl R., Hill M., Heracek J., Bicikova M., Starka L. (2015). Development and validation of LC-MS/MS method for quantification of bisphenol A and estrogens in human plasma and seminal fluid. Talanta.

[61] Hornung R.W., Reed L.D. (1990). Estimation of average concentration in the presence of nondetectable values. Appl. Occup. Environ. Hyg..

